# Supplementary material for: PCM1 is necessary for focal ciliary integrity and is a candidate for severe schizophrenia
Source: Nat Commun. 2020 Nov 19;11:5903. doi: 10.1038/s41467-020-19637-5 (PMC7677393; doi:10.1038/s41467-020-19637-5)
Supplement: Supplementary file 1 — Supplementary Information [file 41467_2020_19637_MOESM1_ESM.pdf]

SUPPLEMENTARY FIGURES:

PCM1 is necessary for focal ciliary integrity and is a candidate for severe schizophrenia

Monroe et al.

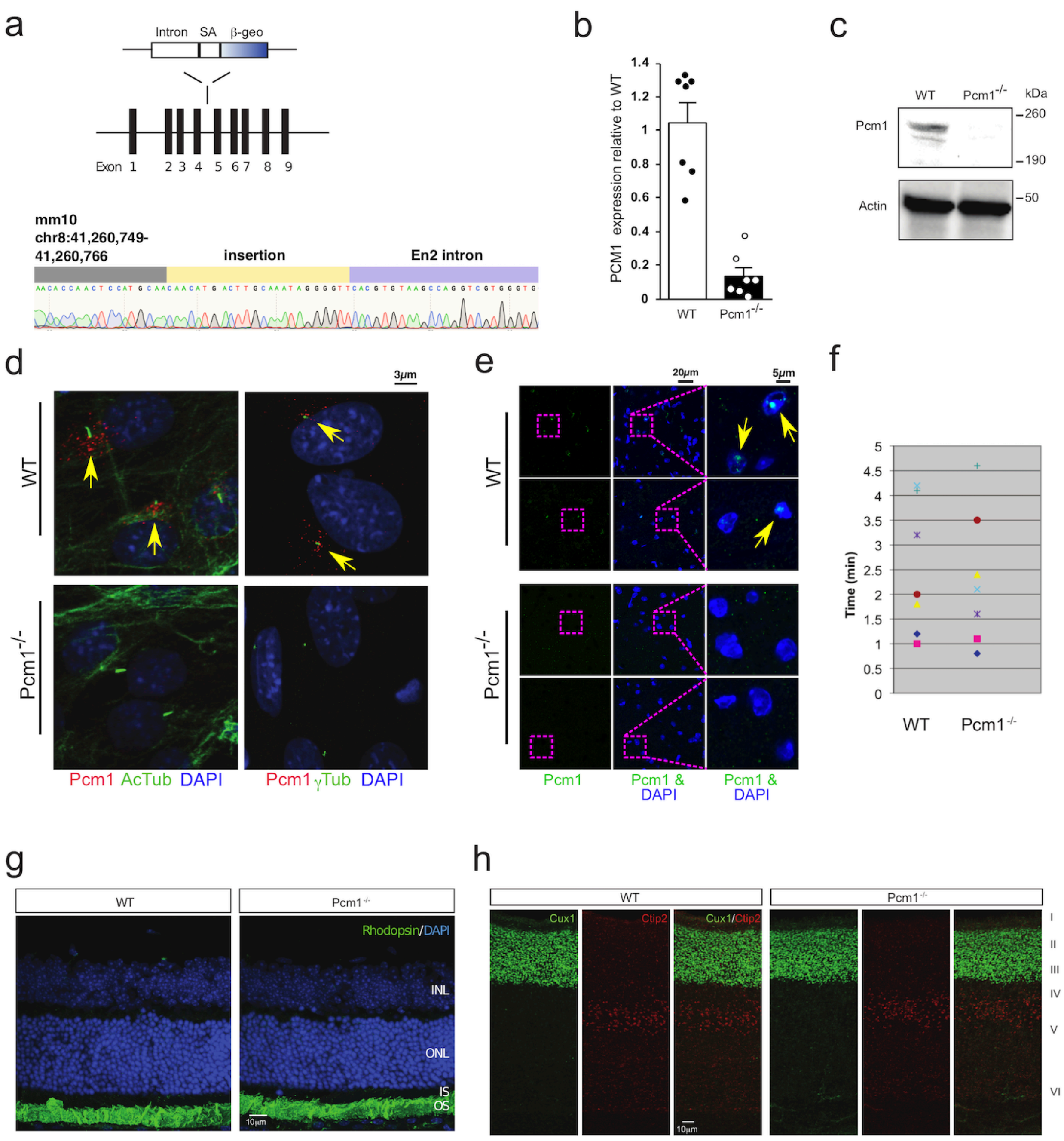
a
Intron
SA
β-geo
Exon 1
2 3 4 5 6 7 8 9
mm10
chr8:41,260,749-
41,260,766
insertion
En2 intron
b
PCM1 expression relative to WT
1.4
1.2
1
0.8
0.6
0.4
0.2
0
WT
Pcm1-/-
c
WT
Pcm1-/-
kDa
Pcm1
260
190
Actin
50
d
3μm
WT
Pcm1-/-
Pcm1 AcTub DAPI
Pcm1 γTub DAPI
e
20μm
5μm
WT
Pcm1-/-
Pcm1
Pcm1 & DAPI
Pcm1 & DAPI
f
Time (min)
5
4.5
4
3.5
3
2.5
2
1.5
1
0.5
0
WT
Pcm1-/-
g
WT
Pcm1-/-
Rhodopsin/DAPI
INL
ONL
IS
OS
10μm
h
WT
Pcm1-/-
Cux1
Ctip2
Cux1/Ctip2
10μm
I
II
III
IV
V
VI

Supplementary Figure 1. Characterization of *Pcm1*$^{-/-}$ mice. a (Top) Gene trap diagram displaying the insertion of the gene trap cassette between exons four and five of the *Pcm1* gene. SA, splice acceptor; β-geo, β-galactosidase/Neomycin cassette. (Bottom) Sanger sequencing confirmation of the precise insertion site. **b** Quantitative RT-PCR analysis of *Pcm1* mRNA from WT and *Pcm1*$^{-/-}$ brain homogenates (N=7; means ± SEM). **c** Immunoblots of cortical brain lysates from WT and *Pcm1*$^{-/-}$ mice. Antibodies against Pcm1 and actin are shown. **d** Primary lung fibroblasts were cultured and stained for Pcm1 and acetylated-tubulin (AcTub) or γ-tubulin (γTub). **e** Representative Pcm1 staining in mouse striatum. **d-e** Arrows indicate the characteristic speckled pattern of Pcm1 localization that is not present in WT cells. **f** Hidden cereal olfactory test (no genotype difference in the time required to find the hidden cereal, N=7). **g** Representative immunohistochemical staining of retinal sections from WT and *Pcm1*$^{-/-}$ mice with rhodopsin and DAPI staining revealing no robust differences between genotypes. **h** Representative staining with Cux1 and Ctip2 show normal cortical lamination in P4 *Pcm1*$^{-/-}$ brain sections. Cortical layers (I to VI) are indicated. Source data and detailed statistical information are provided as a Source Data File

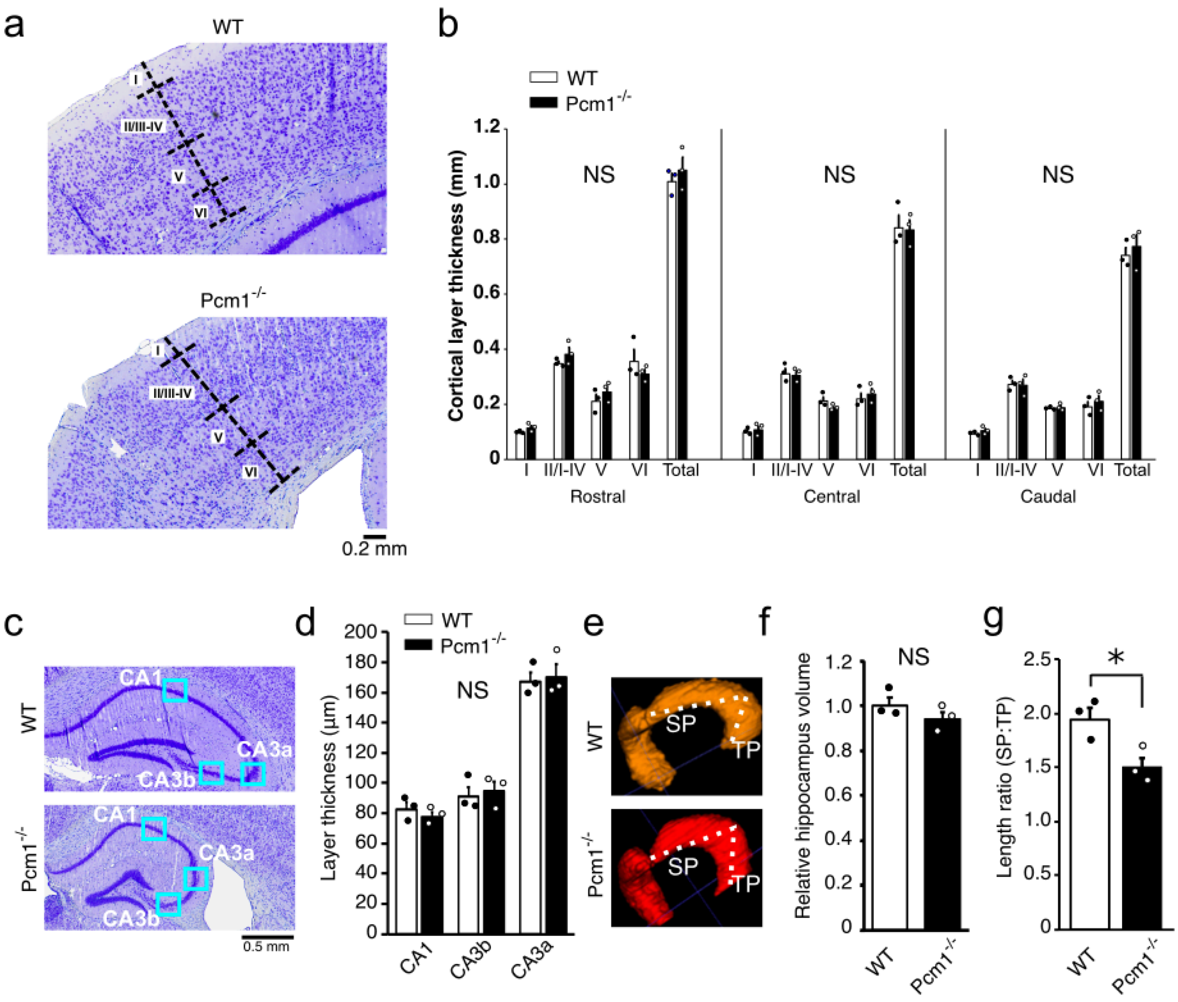

Supplementary Figure 2. Continued neuroanatomical analysis. a Representative Cresyl violet histological sections from WT and *Pcm1$^{-/-}$* cortex showing the cell layers. **b** Cortical layer thickness from different cortical regions (N=4). **c** Representative Cresyl violet histological sections from WT and *Pcm1$^{-/-}$* hippocampus with different regions annotated. **d** Hippocampal layer thickness from the indicated regions in panel c (N=4). **e** Representative volumetric views of the hippocampi, with septal poles (SP) and temporal poles (TP), and their lengths indicated (dots). **f** Relative hippocampal volume (N=3). **g** Ratio of hippocampal septal pole to temporal pole length (N=3; p=0.036). Two-sided t-tests without adjustments. Data presented as means ±SEM; *p<0.05, WT vs. *Pcm1$^{-/-}$*. Source data and detailed statistical information are provided as a Source Data File

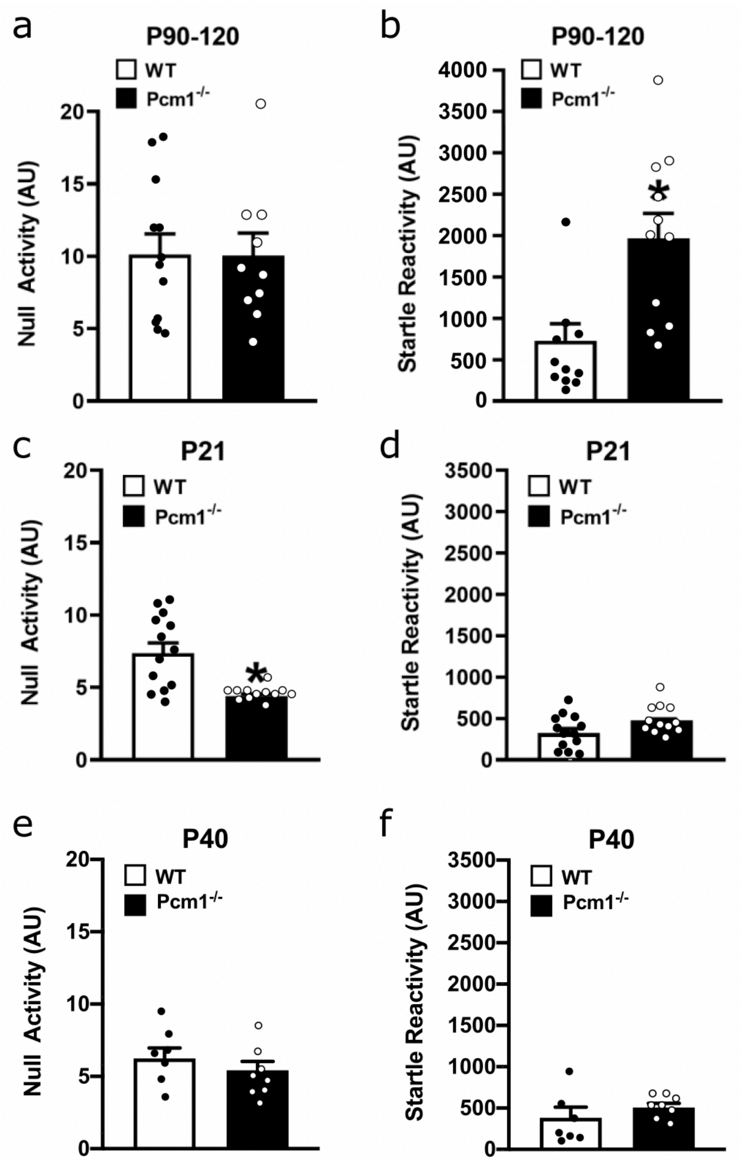

Supplementary Figure 3. Developmental PPI null and startle in *Pcm1*$^{-/-}$ mice. a. Null activity in P90-120 WT and *Pcm1*$^{-/-}$ mice (N=11 and 12 mice/genotype. **b.** Startle activity in P90-120 mice (N=11 and 12 mice/genotype; p=0.003). **c** Null activity in P21 WT and *Pcm1*$^{-/-}$ mice (N=12 and 13 mice/genotype; p<0.001). **d** Startle activity in P21 mice. (N=12 and 13 mice/genotype). **e** Null activity in P40 WT and *Pcm1*$^{-/-}$ (N=7 and 8 mice/genotype**)** **f** Startle activity in P40 mice. mice (N=7 and 8 mice/genotype). a-f Data presented as means ±SEM. Two-sided T-tests without adjustment, *p<0.05, WT vs. *Pcm1*$^{-/-}$. Source data and detailed statistical information are provided as a Source Data File

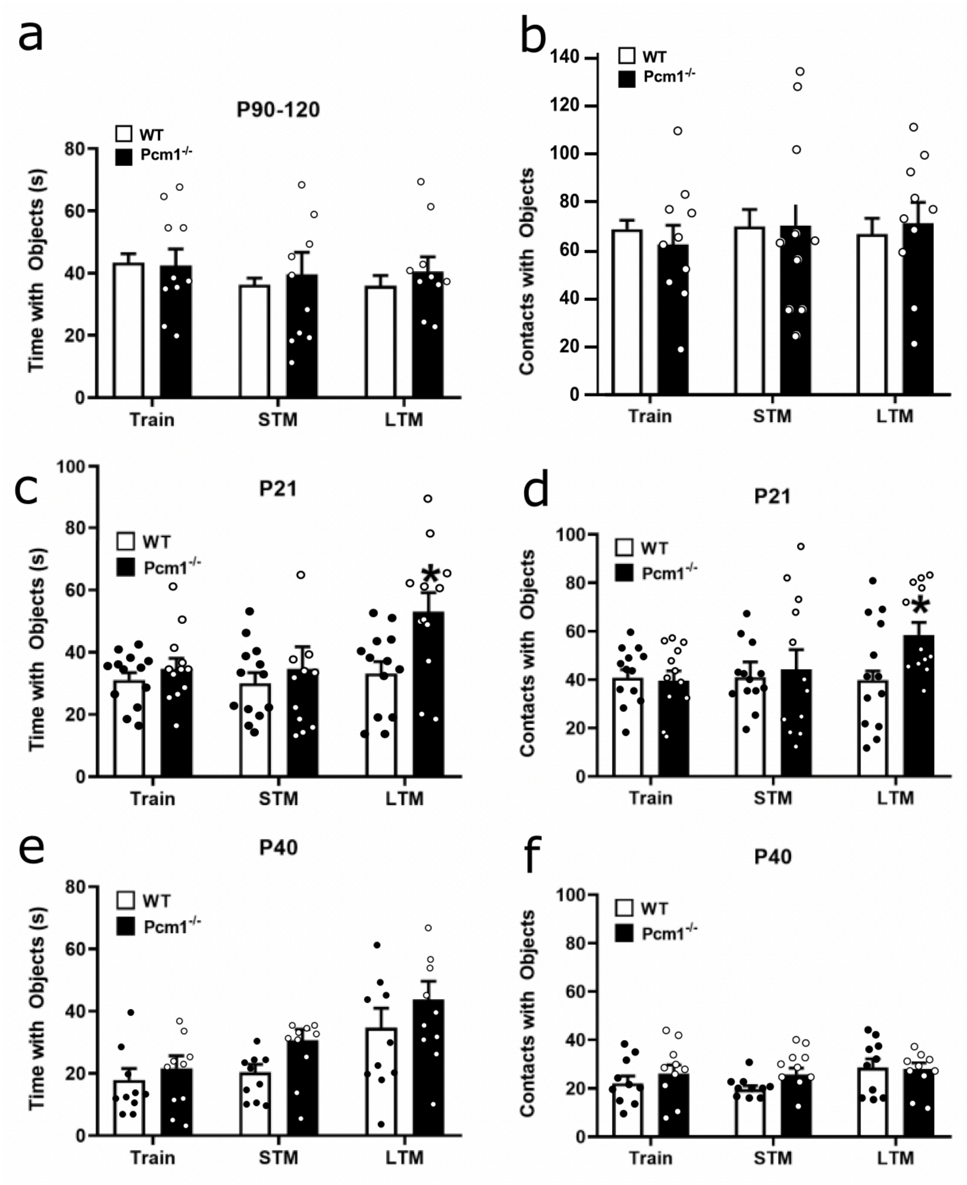

Supplementary Figure 4. Developmental novel object recognition memory numbers and duration of object contacts in *Pcm1*$^{-/-}$ mice. **a** Time with objects for P90-120 WT and *Pcm1*$^{-/-}$ mice mice (N=10 and 14 mice/genotype). **b** Numbers of contacts for P90-120 mice (N=10 and 14 mice/genotype). **c** Time with objects for P21 WT and *Pcm1*$^{-/-}$ mice (N=12 and 13 mice/genotype; p=0.021 (test phase x genotype)). **d** Numbers of object contacts for P21 mice (N=12 and 13 mice/genotype; p=0.007 test phase x genotype). **e** Time with objects for P40 WT and *Pcm1*$^{-/-}$ mice (N=10 mice/genotype; overall main effect of test, p=0.001). **f** Numbers of object contacts for P40 mice (N=10 mice/genotype). Data presented as means ±SEM. RMANOVA, *p<0.05, WT vs. *Pcm1*$^{-/-}$. Source data and detailed statistical information are provided as a Source Data File

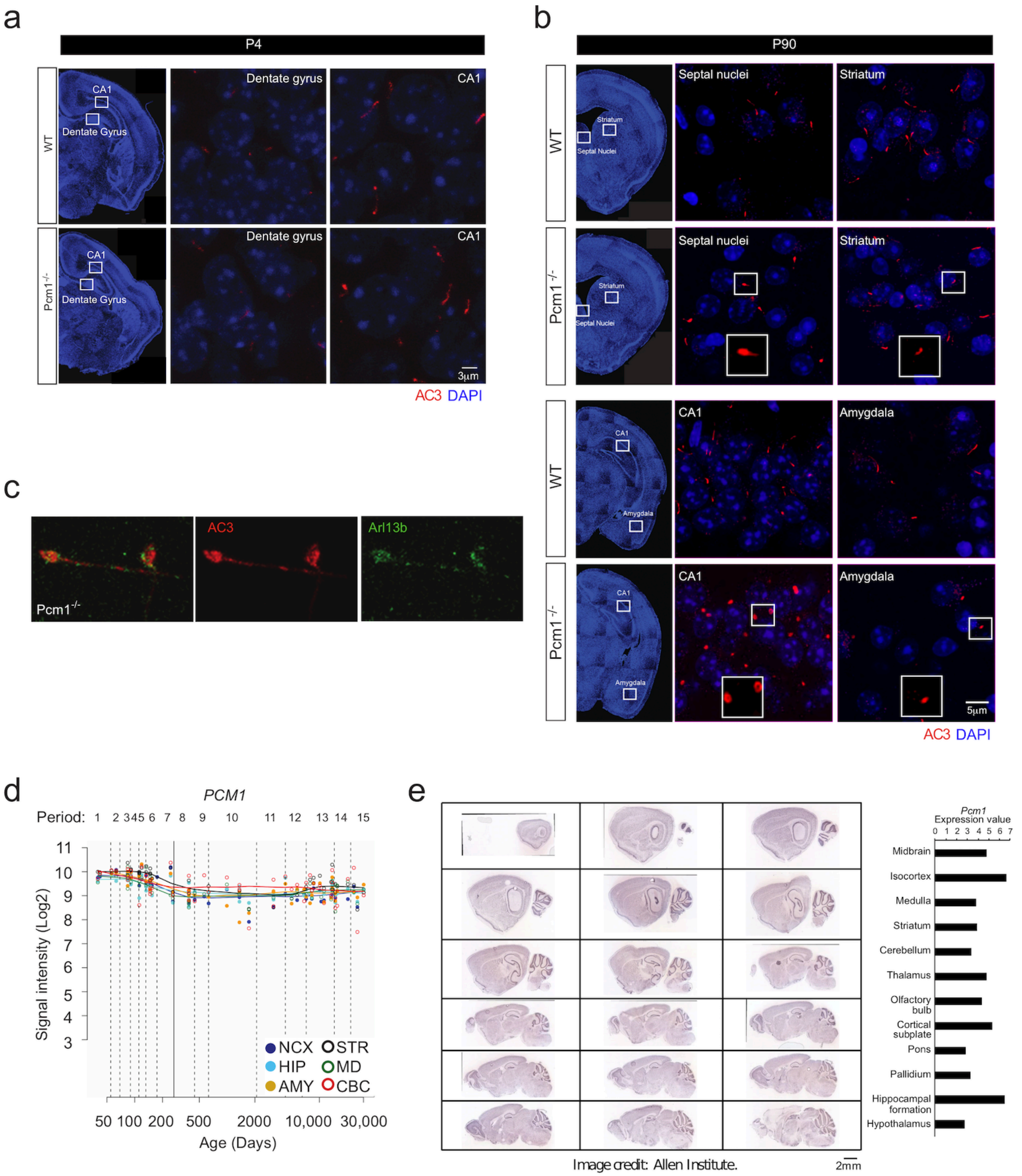
a
P4
WT
Pcm1-/-
CA1
Dentate Gyrus
Dentate gyrus
CA1
3μm
AC3 DAPI
b
P90
WT
Pcm1-/-
Striatum
Septal Nuclei
Septal nuclei
Striatum
CA1
Amygdala
5μm
AC3 DAPI
c
AC3
Arl13b
Pcm1-/-
d
PCM1
Period: 1 2 345 6 7 8 9 10 11 12 13 14 15
Signal intensity (Log2)
11
10
9
8
7
6
5
4
3
NCX
STR
HIP
MD
AMY
CBC
50 100 200 500 2000 10,000 30,000
Age (Days)
e
Image credit: Allen Institute.
2mm
Pcm1
Expression value
0 1 2 3 4 5 6 7
Midbrain
Isocortex
Medulla
Striatum
Cerebellum
Thalamus
Olfactory bulb
Cortical subplate
Pons
Pallidium
Hippocampal formation
Hypothalamus

Supplementary Figure 5. Cilia from WT and *Pcm1*$^{-/-}$ at P4 and in P90 mice; and *PCM1* RNA levels in the human and mouse brain. a Normal cilia morphology in P4 WT and *Pcm1*$^{-/-}$ brain sections. Representative hippocampal regions are shown at low and high magnification. **b** Bulbous cilia in the adult (P90) WT and *Pcm1*$^{-/-}$ brain, with emphasis on the septum, striatum, hippocampus, and amygdala. **c** Both Arl13b and AC3 antibodies stain bulbous cilia with similar intensities suggesting that both proteins localize at similar levels in brain regions from *Pcm1*$^{-/-}$ mice. **d** Profiling for *PCM1* RNA expression during the development of the human brain. Abbreviations: NCX, neocortex; STR, striatum; HIP, hippocampus; MD; AMY, amygdala; and CBC, cerebral cortex. **e** *In situ* hybridizations showing *Pcm1* expression in the adult mouse brain in various regions. Data extracted from the Allen Brain Atlas[45]

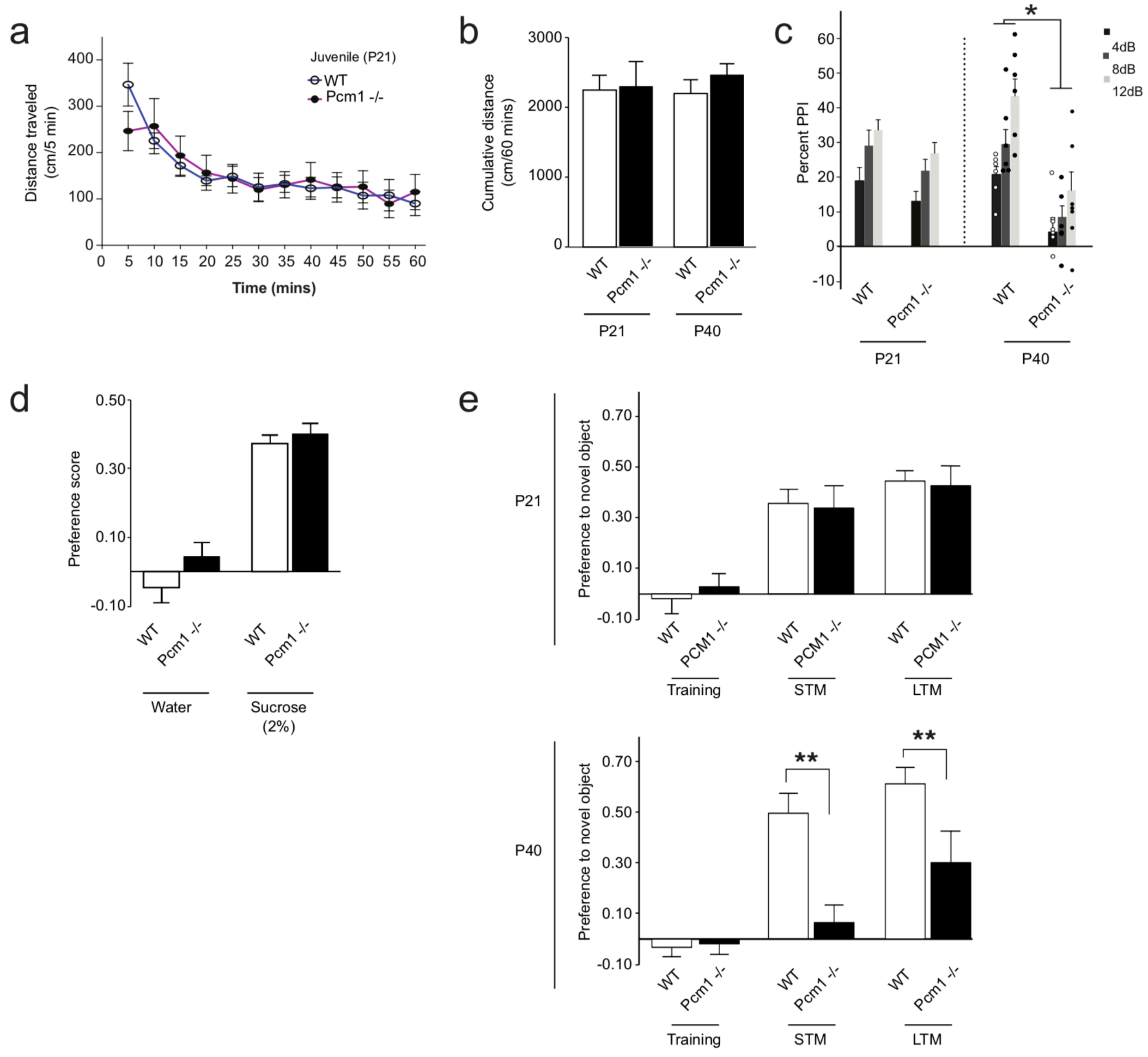

Supplementary Figure 6. Developmental behavioral responses in *Pcm1*$^{-/-}$ mice. a Open field locomotor activity over time in P21 mice (first study, N=7 mice/genotype). **b** Cumulative open field locomotion in P21 mice (a second, replicated experiment of panel a) and in P40 mice (N=12 and 14 mice/genotype). **c** PPI in juvenile (P21-28) and prepubescent (P40) *Pcm1*$^{-/-}$ mice. (P21, N=12 and 13 mice/genotype; P40, N=6 and 7 mice/genotype; p=0.029 intensity x genotype;p<0.001 overall genotype). **d** Anhedonia-like behavior in P40 mice (N=12 and 14 mice/genotype). **e** Performance in the novel object recognition memory test in P21 and P40 mice (P21 N= 13 and 12 mice/genotype; P40, N=10 mice/genotype, p<0.013 test interval x genotype; p<0.001, overall genotype). Data as means ±SEM. RMANOVA, *p<0.05, **p<0.01, WT vs. *Pcm1*$^{-/-}$. Source data and detailed statistical information are provided as a Source Data File

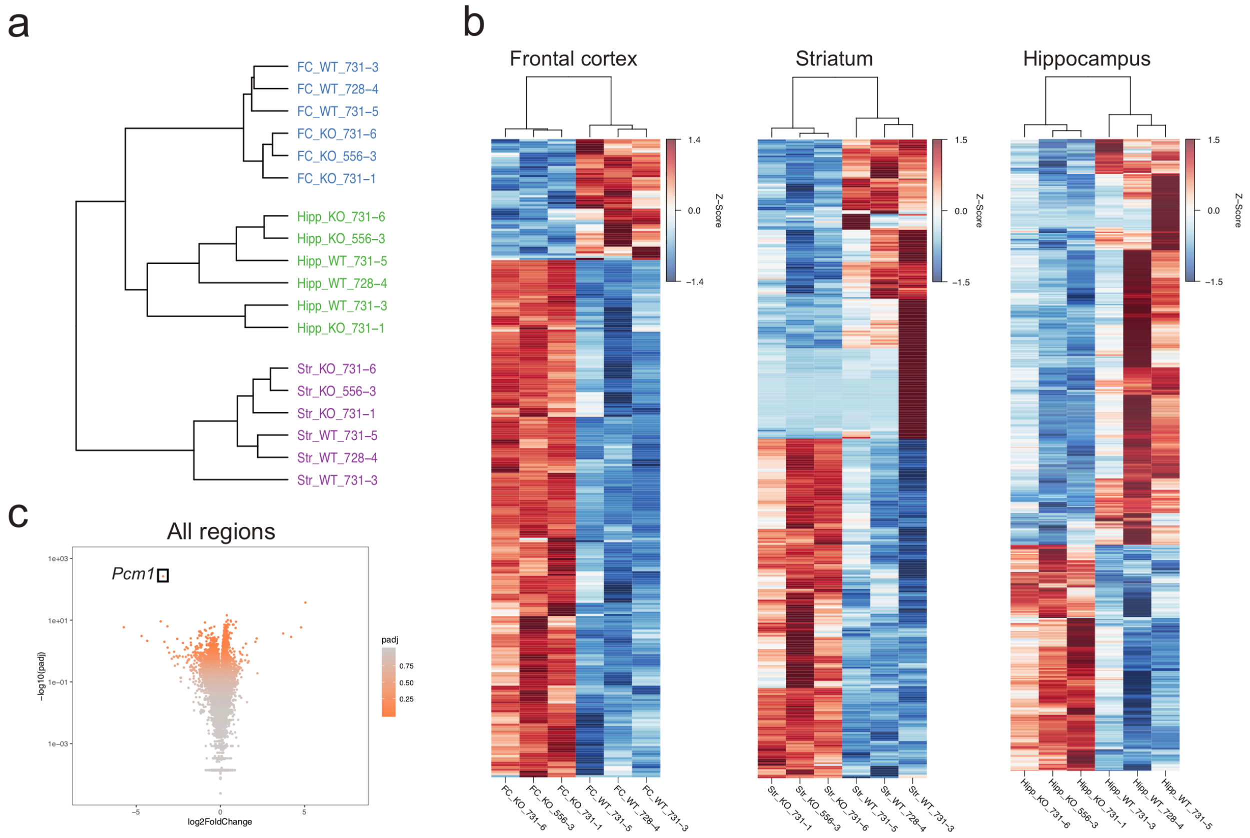

Supplementary Figure 7. RNA-seq heat maps and hierarchical clustering. **a** Hierarchical clustering was performed using the correlation distance with complete linkage of samples. Expression values were normalized and both the samples and genes were clustered using correlation distance with complete linkage. **b** Heat map shows all genes that are differentially expressed (FDR q-value <= 0.05) between each of the conditions. **c** Volcano plot indicating adjusted p-value relative to fold change in the normalized RNA-seq reads when considering all regions in *Pcm1*$^{-/-}$ relative to WT. *Pcm1* is highlighted with a box. RNA-seq data has been made publicly available

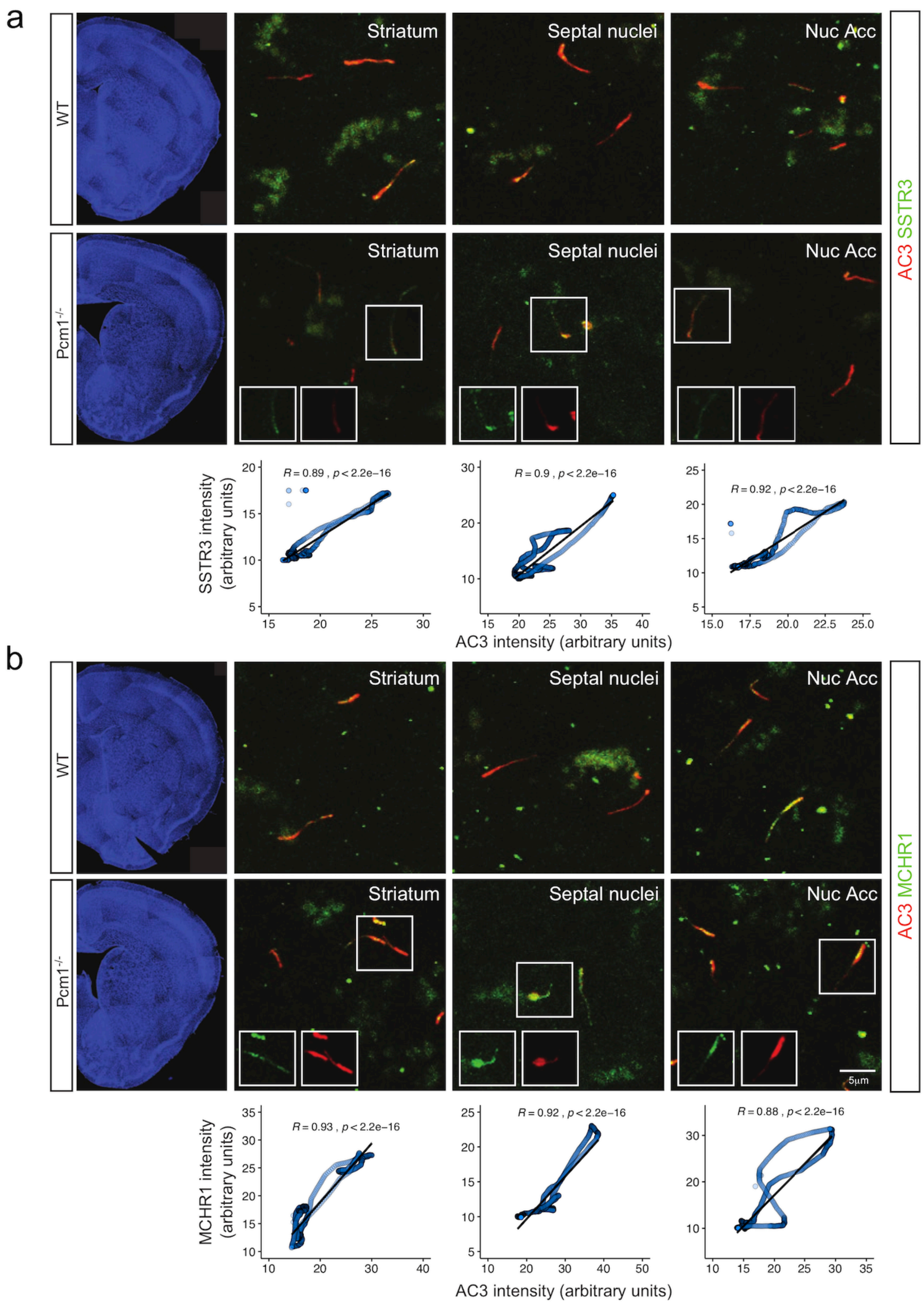
a
WT
Pcm1-/-
Striatum
Septal nuclei
Nuc Acc
AC3 SSTR3
R = 0.89 , p < 2.2e−16
R = 0.9 , p < 2.2e−16
R = 0.92 , p < 2.2e−16
SSTR3 intensity (arbitrary units)
AC3 intensity (arbitrary units)
b
WT
Pcm1-/-
Striatum
Septal nuclei
Nuc Acc
AC3 MCHR1
5μm
R = 0.93 , p < 2.2e−16
R = 0.92 , p < 2.2e−16
R = 0.88 , p < 2.2e−16
MCHR1 intensity (arbitrary units)
AC3 intensity (arbitrary units)

Supplementary Figure 8. Representative cilia stained with AC3, SSTR3, and MCHR1 in brains from WT and *Pcm1*$^{-/-}$ mice. a IHC staining of rostral brain regions for SSTR3 and the cilia (AC3). **b** IHC staining of rostral brain regions for MCHR1 and the cilia (AC3). All *Pcm1*$^{-/-}$ images are shown with intensity quantile-quantile plots (below), along with theoretical reference lines for the indicated regions, confidence intervals as grey shading, and Pearson correlation statistic, with the associated p-values. Source data and detailed statistical information are provided as a Source Data File

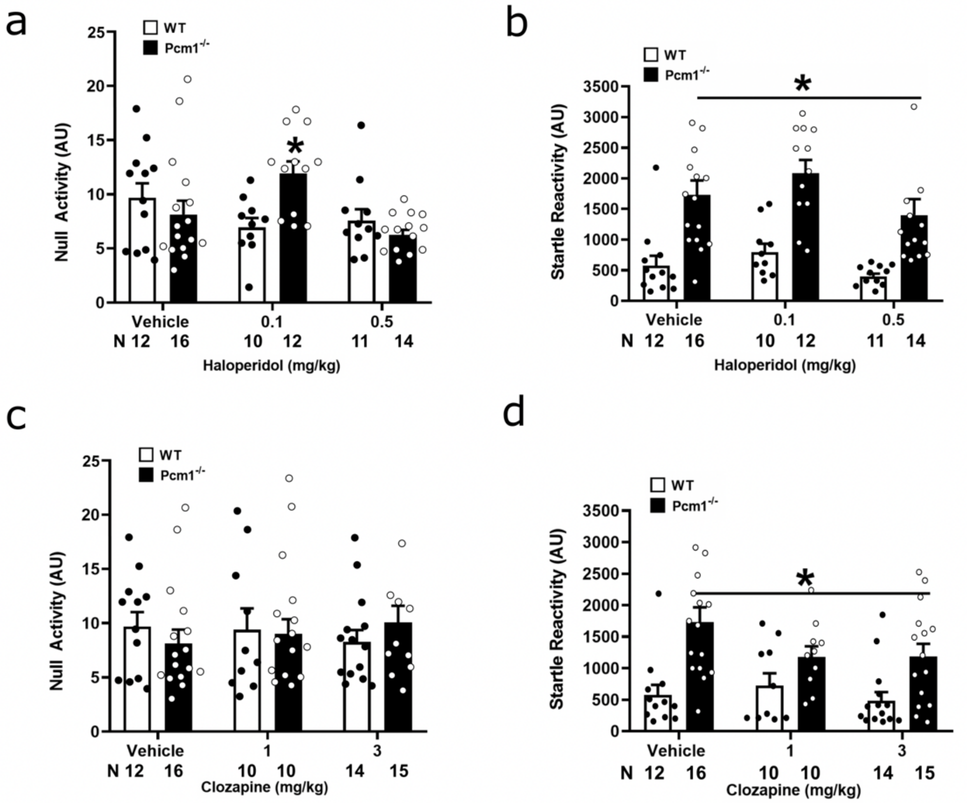

Supplementary Figure 9. PPI null and startle responses in WT and *Pcm1*$^{-/-}$ mice treated with antipsychotic drugs. a Null activity in adult WT and *Pcm1*$^{-/-}$ mice treated with the vehicle, or 0.1 or 0.5 mg/kg haloperidol (N shown in figure mice/genotype, p=0.007 (treatment x genotype)). **b** Startle activity in adult WT and *Pcm1*$^{-/-}$ mice treated with the vehicle or haloperidol (N=10 and 16 mice/genotype, p<0.001 (genotype)). **c** Null activity in adult WT and *Pcm1*$^{-/-}$ mice treated with the vehicle, or 1 or 3 mg/kg clozapine. **d** Startle activity in adult WT and *Pcm1*$^{-/-}$ mice treated with the vehicle or clozapine (N shown in figure; p<0.001 (genotype)). ANOVA, *p<0.05, WT vs. *Pcm1*$^{-/-}$. Source data and detailed statistical information are provided as a Source Data File

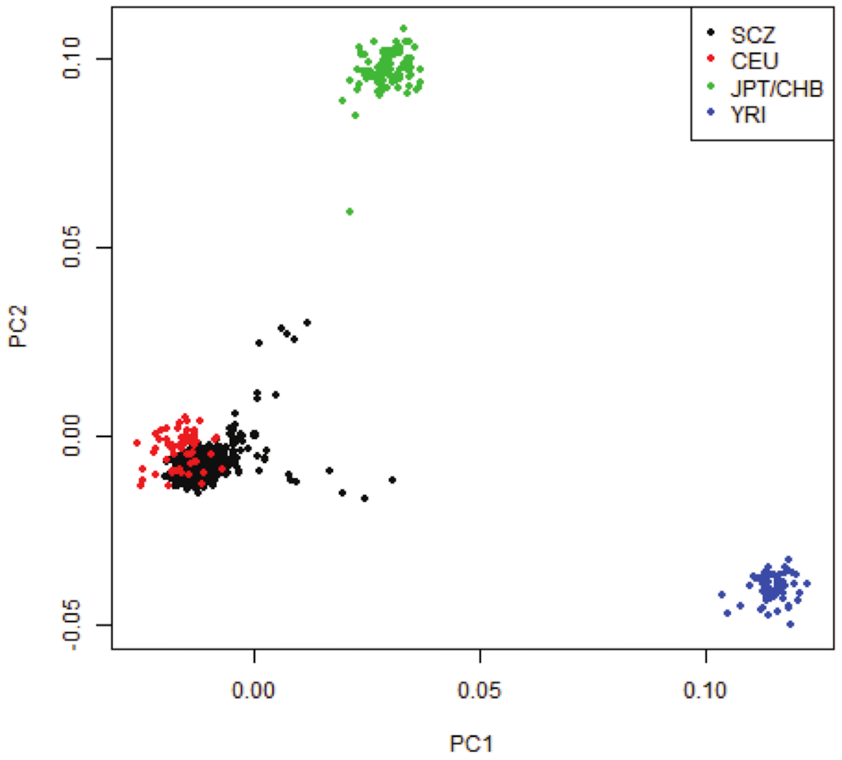

Supplementary Figure 10. Principal component analysis (PCA) plot. An examination of the population substructure among study samples shows that the SZ cohort is most similar to Northern Europeans. Abbreviations: CEU, Utah residents with ancestry from northern and western Europe; YRI, Yoruba in Ibadan, Nigeria; JPT, Japanese in Tokyo, Japan; and CHB, Han Chinese in Beijing, China.

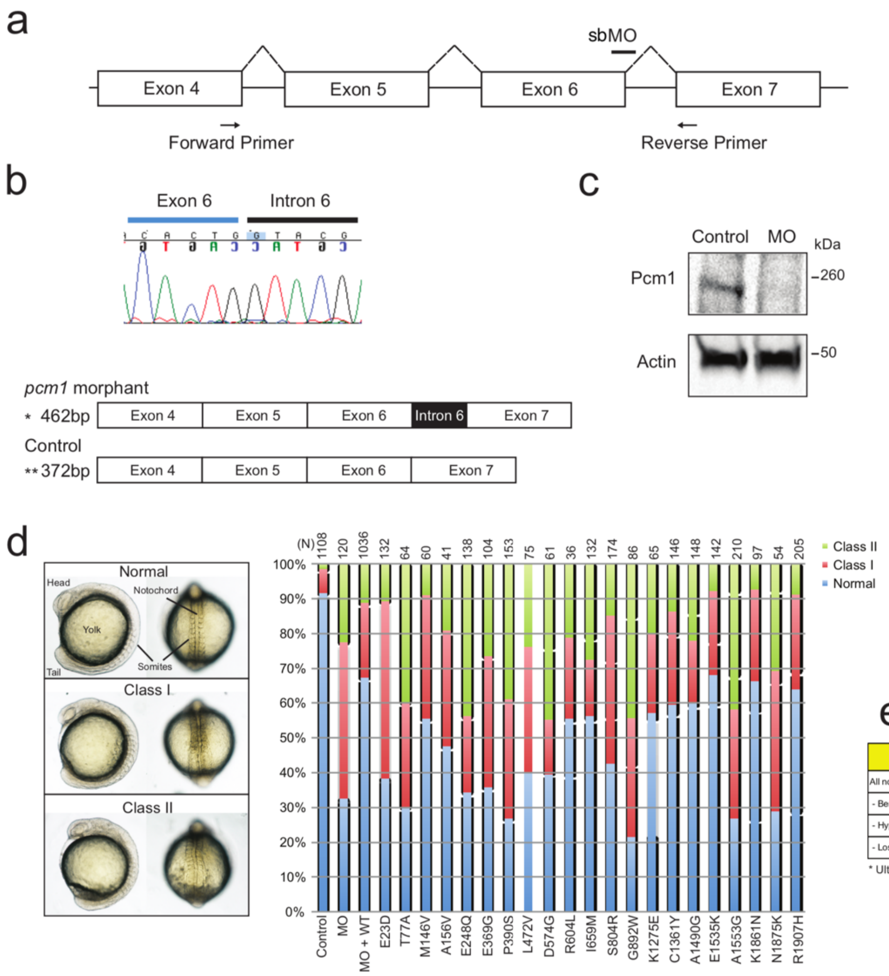

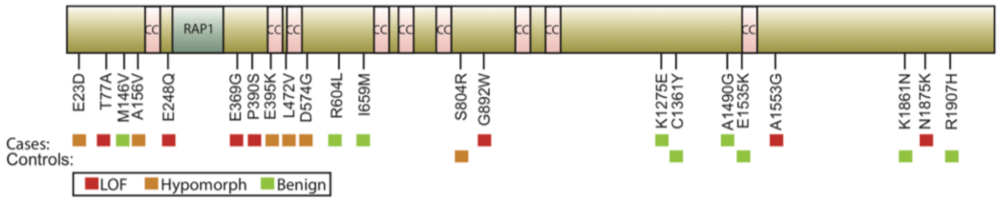

e

Subset	N variants (Cases)	N variants (Controls)	Unweighted *p*-value	Odds ratio
All nonsynonymous variants*	16	5	0.0209	3.007
- Benign	5	4	0.7526	1.219
- Hypomorph	4	1	0.2175	3.016
- Loss of function	7	0	0.0076	15.155

* Ultra-rare = MAF < 0.1%

Supplementary Figure 11. Analysis of *pcm1* suppression in zebrafish and CE phenotypes
a Schematic of the zebrafish *pcm1* gene showing where the splice blocking morpholino (sbMO) targets the 3' aspect of exon 6. **b** Sequencing chromatogram from PCRs after gel purification using forward and reverse primers on cDNA (panel **a**) from 36 hpf wild-type (WT) and MO. The chromatograms are displayed and show a retained intron 6 in the *pcm1* mRNA from MO-injected embryos. **c** Immunoblot showing the effect of the translation-blocking morpholino (tbMO) on zebrafish pcm1 protein levels. **d** Images from zebrafish embryos highlighting normal and two classes of convergence and extension phenotypes. Class II phenotypes are more severe than Class I phenotypes in that the distance between the head and tail is greater and widening of the notochord is more evident. Representative results from convergence and extension phenotypes in zebrafish showing control, MO, or MO + WT human *PCM1* RNA rescue conditions (N shown in figure). **e** Results from gene-based association tests of PCM1 and ASZ using ultra-rare functional variants. Source data and detailed statistical information are provided as a Source Data File

SUPPLEMENTARY TABLES:

Variant	Nucleotide position	Chromosome 8 position (hg19)	*p* -value from MO	*p* -value from MO+WT	N	Functional effect	Allele Frequency (gnomAD)
G6D	G17A	17793136	0.067	<0.0001	43	loss of function	NA
E23D	G69C	17793188	0.0004	0.0007	39	hypomorph	0.000312
T77A	A229G	17794775	0.57	<0.0001	46	loss of function	NA
M146V	A436G	17796342	<0.0001	0.13	40	benign	NA
A156V	C467T	17796373	<0.0001	0.0099	41	hypomorph	0.002916
M200T	T599C	17796505	0.28	<0.0001	40	loss of function	NA
M200I	G600A	17796506	0.0022	0.0049	35	hypomorph	8.13E-06
D214G	A641G	17797228	0.0005	0.0013	42	hypomorph	NA
E248Q	G742C	17797329	0.53	<0.0001	31	loss of function	6.3E-06
E311Q	G931C	17804842	0.0012	0.0036	50	hypomorph	NA
E369G	A1106G	17810513	0.05	<0.0001	41	loss of function	NA
P390S	C1168T	17810575	0.38	<0.0001	29	loss of function	4.23E-06
L472V	C1414G	17813104	0.039	0.0003	39	hypomorph	4.01E-06
G482V	G1445T	17813135	0.0002	0.0012	47	hypomorph	1.2E-05
E543K	G1627A	17814267	<0.0001	0.64	31	benign	7.5E-05
D574G	A1721G	17814847	0.0044	0.0021	39	hypomorph	NA
R604L	G1811T	17815055	<0.0001	0.55	51	benign	NA
E624K	G1870A	17815114	<0.0001	0.58	48	benign	0.001153
I659M	C1977G	17815221	<0.0001	0.62	41	benign	NA
S804R	A2410C	17819630	<0.0001	0.69	48	benign	0.000106
R833T	G2498C	17820644	<0.0001	0.71	45	benign	4.06E-06
C876R	T2626C	17820772	0.0033	0.59	44	benign	8.86E-05
G892W	G2674A	17822097	0.16	0.0007	47	loss of function	4.02E-06
E917G	A2750G	17822077	0.19	<0.0001	30	loss of function	NA
K954N	G2862C	17823514	<0.0001	0.92	30	benign	4.7E-05
N1125S	A3374G	17824641	<0.0001	0.11	39	benign	0.001218
K1275E	A3823G	17830076	<0.0001	0.32	56	benign	0.000729
H1352Y	A4055T	17838211	0.012	0.0045	33	hypomorph	NA
C1361Y	G4082A	17838238	0.0003	0.61	43	benign	4.07E-06
A1490G	C4469G	17847426	<0.0001	0.55	41	benign	2.57E-05
E1535K	G4603A	17849122	<0.0001	0.59	42	benign	NA
A1553G	C4658G	17849177	0.0015	0.0034	48	hypomorph	1.79E-05
G1556D	G4667A	17849186	0.36	<0.0001	42	loss of function	NA
K1861N	A5583C	17871544	<0.0001	0.13	44	benign	4.41E-06
N1875K	T5625G	17872133	0.087	<0.0001	44	loss of function	0.001343
R1907H	G5720A	17872228	<0.0001	0.12	40	benign	6.42E-05
P1913L	C5738T	17872246	0.75	0.0027	43	loss of function	0.0001
A1979S	G5935T	17883053	0.72	0.0027	31	loss of function	0.000228
M597V (Common)	A1789G	17814915	<0.0001	0.62	46	benign	0.733165
A691S (Common)	G2071T	17817553	<0.0001	0.74	34	benign	0.139014

Supplementary Table 1. *PCM1* variants discovered in the severe SZ cohort. Zebrafish results and statistics from variants co-injected with the *pcm1* MO. Genomic loci according to GRCh37 (hg19), allele frequencies retrieved from gnomAD 02/07/2020). Source data and detailed statistical information are provided as a Source Data File

Frontal Cortex

Rank	Pathway
1	Neuropeptide binding
2	Neuropeptide receptor activity
3	Synaptogenesis
4	Hormone activity
5	Amine receptor activity
6	G protein signaling coupled to cyclic nucleotide second messenger
7	G protein coupled receptor activity
8	Cyclic nucleotide mediated signaling
9	Rhodopsin like receptor activity
10	Neurotransmitter binding

Striatum

Rank	Pathway
1	Glutamate receptor activity
2	Amine receptor activity
3	Glutamate signaling pathway
4	Synaptogenesis
5	Protein amino acid n-linked glycosylation
6	Anion transport
7	Transmembrane receptor protein phosphatase activity
8	G protein coupled receptor activity
9	Glycoprotein biosynthetic process
10	Rhodopsin like receptor activity

Hippocampus

Rank	Pathway
1	Neurotransmitter receptor activity
2	Acetylcholine binding
3	Neuropeptide binding
4	Neuropeptide receptor activity
5	Neurotransmitter binding
6	Amine receptor activity
7	Rhodopsin like receptor activity
8	Synaptic transmission
9	G protein coupled receptor activity
10	Transmission of nerve impulse

Supplementary Table 2. Pathway enrichment analysis of WT and *Pcm1*$^{-/-}$ brain regions. Ranking of pathways most significantly altered in the frontal cortex, striatum, and hippocampus from WT and *Pcm1*$^{-/-}$ brains. Source data and detailed statistical information are provided as a Source Data File

Frontal Cortex

Pathway	p-value
Depression	0.042
Schizophrenia	0.004
Mental illness	0.025

Striatum

Pathway	p-value
Depression	0.034
Mental illness	0.013

Hippocampus

Pathway	p-value
Schizophrenia	< 0.001
Mental illness	0.001
Depression	0.037

Supplementary Table 3. Psychiatric disorder gene-set enrichment analysis on genes altered in *Pcm1*$^{-/-}$ brains. Comparison of mutant mouse transcriptomes with expression profiling of genes found to be dysregulated in human psychiatric disorders. Source data and detailed statistical information are provided as a Source Data File
